# Eicosapentaenoic Acid Enhances Heat Stress-Impaired Intestinal Epithelial Barrier Function in Caco-2 Cells

**DOI:** 10.1371/journal.pone.0073571

**Published:** 2013-09-16

**Authors:** Guizhen Xiao, Liqun Tang, Fangfang Yuan, Wei Zhu, Shaoheng Zhang, Zhifeng Liu, Yan Geng, Xiaowen Qiu, Yali Zhang, Lei Su

**Affiliations:** 1 Guangdong Provincial Key Laboratory of Gastroenterology, Department of Gastroenterology, Nanfang Hospital, Southern Medical University, Guangzhou, China; 2 Key Laboratory of Hot Zone Trauma Care and Tissue Repair of PLA, Department of Intensive Care Unit, General Hospital of Guangzhou Military Command, Guangzhou, China; 3 Department of Nutrition, General Hospital of Guangzhou Military Command, Guangzhou, China; Universidade Federal do Rio de Janeiro, Brazil

## Abstract

**Objective:**

Dysfunction of the intestinal epithelial tight junction (TJ) barrier is known to have an important etiologic role in the pathophysiology of heat stroke. N-3 polyunsaturated fatty acids (PUFAs), including eicosapentaenoic acid (EPA) and docosahexaenoic acid (DHA), play a role in maintaining and protecting the TJ structure and function. This study is aimed at investigating whether n-3 PUFAs could alleviate heat stress-induced dysfunction of intestinal tight junction.

**Methods:**

Human intestinal epithelial Caco-2 cells were pre-incubated with EPA, DHA or arachidonic acid (AA) and then exposed to heat stress. Transepithelial electrical resistance (TEER) and Horseradish Peroxidase (HRP) permeability were measured to analyze barrier integrity. Levels of TJ proteins, including occludin, ZO-1 and claudin-2, were analyzed by Western blot and localized by immunofluorescence microscopy. Messenger RNA levels were determined by quantitative real time polymerase chain reaction (Q-PCR). TJ morphology was observed by transmission electron microscopy.

**Results:**

EPA effectively attenuated the decrease in TEER and impairment of intestinal permeability in HRP flux induced by heat exposure. EPA significantly elevated the expression of occludin and ZO-1, while DHA was less effective and AA was not at all effective. The distortion and redistribution of TJ proteins, and disruption of morphology were also effectively prevented by pretreatment with EPA.

**Conclusion:**

This study indicates for the first time that EPA is more potent than DHA in protecting against heat-induced permeability dysfunction and epithelial barrier damage of tight junction.

## Introduction

The intestinal epithelial barrier prevents the entry of luminal pathogens such as bacteria and endotoxins into blood circulation. As the most important component of the epithelial barrier, a functional tight junction (TJ), which forms a continuous circumferential seal at the apical-most portion of cells, is required for the maintenance of the barrier function of the intestine [1], [2]. TJ breakdown contributes to intestinal hyperpermeability. Moreover, bacterial products from the intestinal lumen entering into the circulation cause systemic inflammatory response syndrome and multiple organ failure [3]. Heat stroke is a severe illness characterized by an elevated core body temperature above 40°C and central nervous system dysfunction associated with mucosal damage [4]. Intestinal barrier function plays an important role in the pathophysiology of heat stroke in rat models, and the Caco-2 and T84 cells monolayer exposed to heat stress. Heat stress impairs the intestinal barrier integrity by increasing intestinal permeability and reducing epithelial resistance [5], [6]. It has been reported that intestinal permeability to endotoxin or lipopolysaccharides (LPS) from the gut entering the circulation increases in heat-stressed animals [7], [8]. On the contrary, anti-LPS antibodies protect against the transition from heat stress to heatstroke [9]. Therefore, protecting the integrity of the intestinal barrier is an important goal in the prevention of heatstroke.

Previous studies have found that the supplementation of n-3 fatty acids (PUFAs), which include eicosapentaenoic acid (EPA, 20:5 n-3) and docosahexaenoic acid (DHA, 22:6 n-3), effectively prevents the disruption of TJ structure, the decrease of transepithelial electrical resistance (TEER) and the elevation of flux of FITC-dextran (FD4) induced by inflammatory factors [10], [11]. N-3 PUFAs have shown potential beneficial effects on the immune response in experimental models of rheumatoid arthritis, inflammatory bowel disease and psoriasis by down-regulating the production of pro-inflammatory signals and supporting the intestinal barrier [12]. The evidence demonstrates that EPA up-regulates the expression of TJ protein occludin, but arachidonic acid (AA, 20∶4 n-6) exerts an down-regulatory effect in endothelial cells [13]. Moreover, it has also been demonstrated that PUFAs down-regulate endotoxin translocation from the gut into the systemic circulation in rat models [14].

In spite of these findings, the individual effects of n-3 PUFAs on TJ in intestinal epithelial cells to heat exposure have not been investigated. We therefore hypothesized that the supplementation of n-3 PUFAs before heat exposure would reverse the heat stress-related increase in TJ permeability and reorganization of the TJ proteins. This would improve organ function by protecting the gut barrier and decreasing plasma endotoxin levels. In this study we examined the effect of n-3 fatty acids on the effects of heat stress-induced dysfunction of the intestinal epithelial barrier in Caco-2 monolayers. Caco-2 cells were used as a model to form typical TJ structure similar to mature intestinal epithelium in vitro [15].

## Materials and Methods

All PUFAs were purchased from Sigma-Aldrich (St. Louis, MO). PUFAs of the n-3 series were: eicosapentaenoic acid (EPA) and docosahexaenoic acid (DHA,). The control was arachidonic acid (AA). Ascorbic acid (vitamin C) and alpha-tocopherol (vitamin E) were from Sigma-Aldrich (St. Louis, MO). Antibodies used for these experiments were mouse anti-occludin (BD Biosciences, Franklin Lakes, NJ), rabbit anti-ZO-1 (Invitrogen, Camarillo, CA) and mouse anti-claudin-2 (Invitrogen, Camarillo, CA).

### Cell culture

Caco-2 cells (ATCC, Manassas, VA) were grown as a monolayer in DMEM media supplemented with 10% heat inactivated fetal bovine serum (FBS) (GIBCO) at 37°C in a humidified atmosphere of 5% CO_2_. Upon about 90% confluence, cells were split using 0.05% trypsin plus 1 mM EDTA.

### Preparation and treatment of PUFAs used in experiments

PUFAs were diluted in 100% ethanol to a stock concentration of 400 mM at −80°C. Final PUFA concentrations in the culture medium were 50 μM, with vitamin C and vitamin E at final concentrations of 75 μM and 20 μM respectively (also present in the control group without PUFA).

In the experimental group, EPA, DHA or AA was added to the cells on the second day of culture. Monolayer of Caco-2 cells pre-incubated with PUFAs (50 μM) for 96 h. In the control group, the medium consisting of only the PUFAs solvent (1∶8000 ethanol) was used to ensure the same concentration of ethanol in all groups. Medium and additives were changed every 24 h. For each PUFA studies, control experiments consisted of administration of the PUFA solvent (1∶8000 ethanol) were performed.

### Measurement of transepithelial electrical resistance (TEER)

2.0×10^6^ Caco-2 cells per well were seeded on the collagen-coated membrane transwell inserts (6.5 mm diameter inserts, 3 μm pore size; Corning, USA) with 200 μL culture medium added to the apical chamber and 600 μL to the basolateral chamber. The electrical resistance of confluent polarized Caco-2 monolayers was measured by TEER with an electrical resistance system (EVOM; World Precision Instruments, Berlin, Germany). A pair of chopstick electrodes was placed at each of the apical and basolateral chambers of three different points to evaluate TEER. Readings were taken every 24 h until the net TEER had risen steadily above 250 Ω cm^2^ (at days 7–14). At this point, experiments were carried out. In experiments involving temperature changes, TEER measurements were performed prior to and after heat stress. In experiments using PUFAs, the PUFAs were added to both the apical and the basolateral chamber. TEER measurements were performed prior to the change of medium at 0 h, 24 h, 48 h, 72 h and 96 h of incubation and after heat stress.

### Intestinal paracellular permeability assay

Intestinal paracellular permeability across cell monolayers was determined by measuring the flux of Horseradish peroxidase (HRP, type V; Sigma). HRP (3.4×10^–6^ mol/L) was added to medium in the apical chamber of transwells. After exposure to heat stress for 1h, samples were carefully taken from basolateral chambers and assayed for HRP by TMB Horseradish Peroxidase Color Development Solution for ELISA(Beyotime, China). Enzyme activity was determined from the rate of increase in optical density at 370 nm.

### Western blotting analysis

Caco-2 monolayers were cultured and then harvested 24 hours after 1 h of heat exposure. Protein extracts of whole cells and extraction of membrane-bound and cytosolic fractions by Membrane and Cytosol Protein Extraction Kit (Beyotime, China) were subjected to Western Blotting. Protein concentration was assessed by a BCA protein assay kit. Equal amounts of protein for each sample was separated by SDS-PAGE through a 10% acrylamide gel and transferred to polyvinylidene difluoride transfer membranes (Millpore, Bedford, MA). Membranes were blocked for 2 h at room temperature with 5% non-fat dried milk in TBS containing 0.05% Tween-20 buffer and incubated overnight at 4°C with anti-occludin (1∶500), anti-ZO-1 (1∶200) or anti-claudin-2 (1∶200). After washing three times for 5 min in TBST buffer, the membranes were incubated with the secondary antibodies for 2 h at room temperature. Protein bands were detected with Immobilon Western (Millipore Corporation, Billerica, USA) and analyzed with the Bio-Image Analysis System (Syngene, Frederick, USA).

### Real-time quantitative PCR analysis

Caco-2 monolayers were cultured 24 hours after 1 h of heat exposure. Total RNA was extracted from the cultured cells following the manufacturer's instructions of Trizol isolation (TaKaRa Bio, Japan). RNA was reverse-transcribed to cDNA using PrimeScript RT reagent kit with gDNA Eraser (Takara, China). The PCR mixture (20 μl final volume per reaction) was prepared as described by the manufacturer. Amplifications were performed by quantitative real-time RT-PCR using SYBR Green I Maser kit (Roche, Germany) under the following conditions: 45 cycles of 95°C for 10 s, 60°C for 20 s, then 72°C for 30 s on LightCycler 480 II (Roche, Rptkreuz, SWI). Specific primers were for Glyceraldehyde-3-phosphate dehydrogenase (GAPDH) (Forward) 5′- GAA GGT GAA GGT CGG AGT-3′ and (Reverse) 5′-GAA GAT GGT GAT GGG ATT TC-3′,for occluding (Forward) 5′- CCC ATC TGA CTA TGT GGA AAG A-3′ and (Reverse) 5′- AAA ACC GCT TGT CAT TCA CTT TG-3′, for ZO-1 (Forward) 5′-CGG TCC TCT GAG CCT GTA AG-3′ and (Reverse) 5′-GGA TCT ACA TGC GAC GAC AA-3′. GAPDH was used as the endogenous reference gene to normalize the data.

### Immunostaining of TJ proteins

Caco-2 monolayers were cultured 24 hours after 1 h of heat exposure. Caco-2 cells on coverslips were washed twice in PBS and were fixed with methanol for 15 min. After being made permeable with 0.5% Triton X-100 in PBS at room temperature for 10 min, cells were blocked with 5% bovine serum albumin in PBS for 1 h. The Caco-2 monolayers were incubated with primary antibodies (1∶50) overnight at 4°C. After being washed with PBS, cells were incubated sequentially with DyLight-TFP Ester secondary antibody (1∶100) for 1 h at room temperature. TJ proteins were visualized and images were obtained under a fluorescence microscope (OLYMPUS BX51, Japan).

### Transmission electron microscopy

Caco-2 monolayers were cultured 24 hours after 1 h of heat exposure. Cells were washed twice in PBS and fixed in 2.5% glutaraldehyde in 0.1 M sodium cacodylate buffer overnight at 4°C. After three washes in PBS buffer, the cells were suspended in 2.5% glutaraldehyde and osmium tetroxide and fixed for 1 hour. Then, the cells were suspended in 1% uranyl acetate for 2 hour. After dehydration in acetone, the cells were embedded in an acetone/plastic mixture and polymerized at 65°C for 48 h. Finally, ultrathin sections were cut and stained. Then, sections were viewed and images were captured by transmission electron microscopy (HITACH H-7650, Japan).

### Fatty acid analysis

After 96 h of supplementation with PUFAs, the cells were subjected to fatty acid analysis performed according to the previous method [16]. The fatty acids of all cellular lipids were extracted using a chloroform/methanol mixture in a 2∶1 ratio containing 0.005% butylated hydroxytoluene. They were then methylated by 14% BF3/methanol reagent for 1 h. Methyl esters of the fatty acids were quantified by Gas Chromatography-Mass Selective Detector (HP 6890–5973, Agilent, USA) with a capillary column (30 m ×250 µm ×0.25 µm). The initial temperature was 75°C and then increased to 120°C and maintained for 10 min, then maintained at 150°C for 10 min, and finally at 250°C for 1 min. Fatty acid compositions were expressed as compensated area normalization [17].

### Statistical analysis

Sigmastat statistical software (SPSS 13.0, Chicago, IL) was used to analyze results. All data are expressed as means ± SD. Statistical significance of differences was determined with one-way analysis of variance (ANOVA) among all treatment groups. A two-tailed *P*<0.05 was used to indicate statistical significance.

## Results

### Increasing temperature induces intestinal epithelial barrier disruption

Epithelial barrier integrity and paracellular permeability were determined by the measurement of TEER and flux of HRP. Since basal resistance slightly differed in independent wells, the data are presented relative (% TEER) to baseline (before heat exposure  = 1).

**Figure 1:**
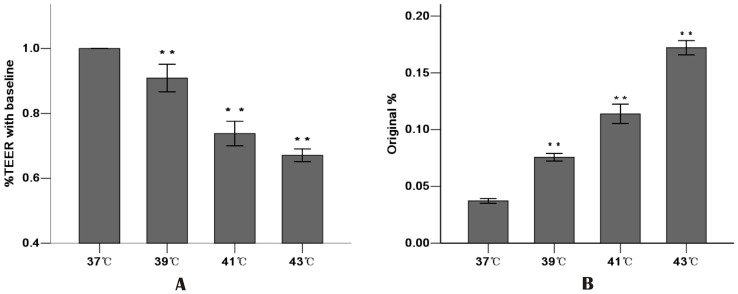
Effect of increasing temperature on Caco-2 monolayer barrier function. Caco-2 monolayers were exposed to increasing temperature for 1 h from 37°C to 43°C. **A:** Increasing temperature decreased TEER. TEER was recorded before (used as a baseline) and after heat stress. TEER was presented relative (%TEER) to baseline. **B:** Increasing temperature increased HRP flux. The amount of HRP in the basolateral chambers was expressed as a percentage of added HRP (original%). Values are means ± SD. ** P<0.01, compared with 37°C group. N = 6 per group.

Increasing the temperature resulted in the reduction of TEER. The higher the temperature, the lower the TEER in the Caco-2 monolayer cells. Compared with the 37°C group (1.04±0.06), increasing the temperature to 39°C showed a decrease in TEER (0.91±0.04, P<0.01). The 41°C group and the 43°C group showed dramatic and significant drops in TEER (0.74±0.04 and 0.67±0.02, respectively, compared with the 37°C group, P<0.01) (**Fig. 1A**).

The permeability for HRP into the basolateral chambers, which was determined by the calculated flux, was expressed as a percentage of added HRP marker. The significant increase in paracellular permeability of HRP flux was accompanied by the reduction in TEER. Increasing temperature also correlated with a significant increase in HRP flux. Compared with the 37°C group, HRP flux increased 1.7 fold in the 39°C group, 2.6 fold in the 41°C group and 3.9 fold in the 43°C group **(** **Fig. 1B** **).** These results indicated that increasing temperature significantly weakened the intestinal epithelial barrier function related to the drop in TEER and the increase in HRP permeability.

### Increasing temperature regulates expression of TJ proteins

**Figure 2:**
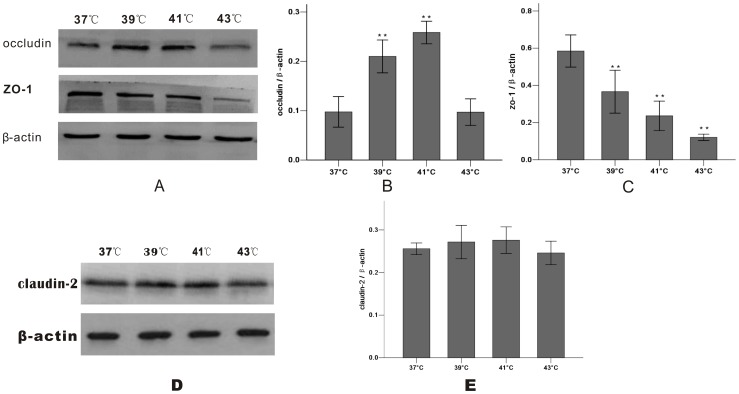
Temperature-course effect of heat exposure (37°C – 43°C) for 1 h on TJ protein expression in Caco-2 monolayers. Samples were harvested 24 hours after 1 h of heat exposure and analyzed by Western blotting (**A**, **D**). **B**: Heat exposure caused a significant increase in expression of occludin, but decrease was observed when exposed to 43°C. **C**: The exposure to heat produced a progressive decrease in ZO-1 protein expression. **E**: Level of claudin-2 protein in total cell extract was not affected by heat exposure. Results were reported as means ± SD from 3 independent experiments. Values were normalized to β-actin. * P<0.05, ** P<0.01 compared with 37°C group.

Cells were exposed to designated temperatures (from 37°C to 43°C) for 1 h. The expression of TJ proteins with increasing temperature was examined by Western blotting analysis. The expression of occludin increased from 37°C to 41°C and reached maximal levels at 41°C. However, occludin expression decreased at 43°C compared with that at 41°C. The expression of ZO-1 protein decreased as the temperature rose and no markedly change in claudin-2 (**Fig. 2**).

**Figure 3:**
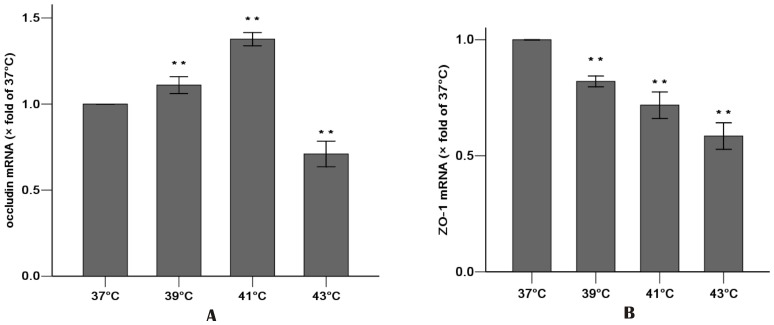
Effect of increasing temperature (37°C – 43°C) for 1 h on the gene expressions of occludin (A) and ZO-1 (B) by Real-time PCR. Cells were cultured for 24 h after 1 h heat exposure. Values were normalized to 37°C group (37°C set to 1). Results were reported as means ± SD. N = 3 per group. ** P<0.01 compared with 37°C group.

Real-time PCR showed the effects on expression of mRNA. Values were normalized to the 37°C group (37°C set to 1). Heat exposure (from 37°C to 41°C) resulted in a progressive increase in occludin mRNA expression, which then decreased at 43°C (**Fig. 3A**). The heat exposure also resulted in a significant decrease in ZO-1 mRNA expression (**Fig. 3B**).

### EPA reduces high temperature impaired permeability

**Figure 4:**
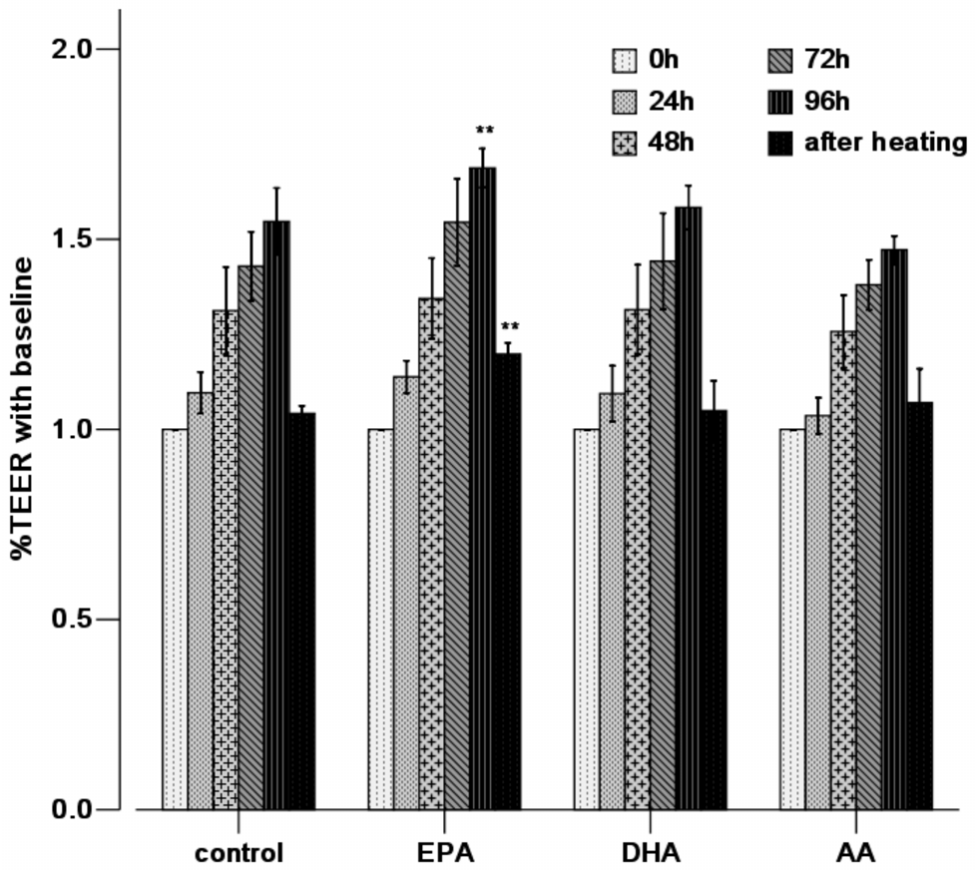
EPA enhances epithelial barrier integrity and ameliorates heat-induced barrier disruption by measuring TEER. Caco-2 monolayers were treated with heat at 43°C for 1 h after absence (control) or presence of PUFAs for 96 h. TEER measurements were performed at 0, 24, 48, 72 and 96 h of incubation and after heat stress. TEER was presented as percentage (%TEER) of initial resistance (baseline  = 1). Values are means ± SD. N = 6 per group. * P<0.05, ** P<0.01 compared with control at same time point.

Confluent Caco-2 cell groups with PUFA (50 μM) pre-incubation for 96 h were exposed to heat stress of 43°C for 1 h. Compared with the control group (1.54±0.08), the TEER at 96 h was significantly increased in the EPA group (1.69±0.05, *P*<0.01), while there were no significant differences at any time points (0–96 h) after incubation in other groups. After 1 h of 43°C heat stress, there was a significant decrease in TEER in the Caco-2 monolayer cells. EPA prevented the decrease of TEER induced by heat stress (1.20±0.03 vs. 1.04±0.02, *P*<0.01 compared with the control group), while DHA and AA do so to a lesser extent (**Fig. 4**).

**Figure 5:**
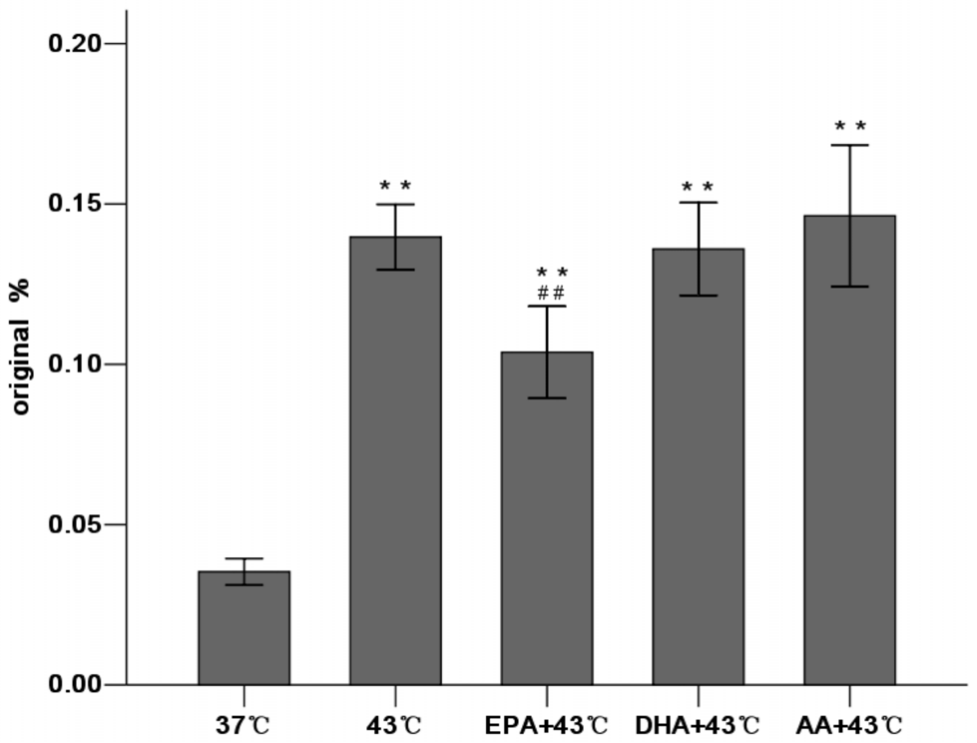
EPA decreases paracellular permeability induced by heat stress by determining HRP flux. Caco-2 monolayers were treated with heat at 43°C for 1 h after absence (control) or presence of PUFAs for 96 h. HRP transport in the basolateral chambers was calculated as a percentage of added HRP after heat stress,. Values are means ± SD. N = 6 per group. * P<0.05, ** P<0.01 compared with 37°C group. ^#^ P<0.05,^ # #^P<0.01 compared with 43°C group.

Our results found that EPA reversed the increase of paracellular permeability induced by heating (0.099±0.004 vs. 0.139±0.004, *P*<0.01 compared with the 43°C group). However, HRP flux remained at high levels in the DHA and AA groups (0.134±0.005 and 0.148±0.010 respectively) (**Fig. 5**). These results indicate that only EPA pretreatment could reinforce TJ function and reverse the increased TJ permeability induced by heat stress, while DHA and AA could not.

### EPA prevents distortion of TJ proteins induced by heat stress

**Figure 6:**
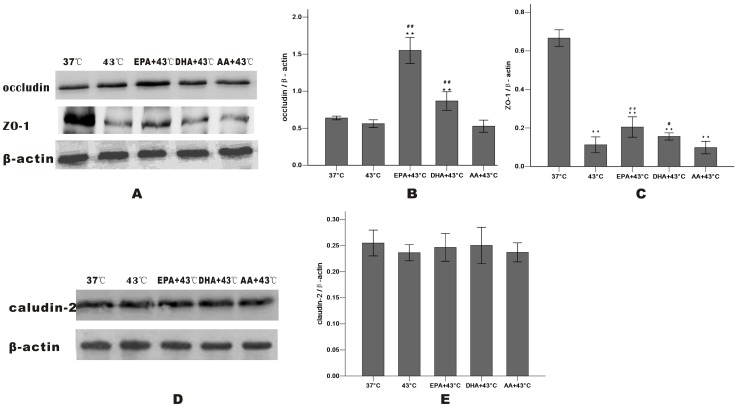
Effect of PUFAs on heat-induced change in protein expression of whole cells by Western blot analysis. Caco-2 monolayers were cultured for 24 h after 1 h of heat exposure without (37°C group and 43°C group) or with PUFAs pre-incubation for 96 h. TJ proteins were shown **(A, D)**: occludin **(B)**, ZO-1 **(C)** and claudin-2 **(E)**. Results were reported as means ± SD from 3 independent experiments. Values were normalized to β-actin. * P<0.05, ** P<0.01 compared with 37°C group.^ #^ P<0.05, ^##^ P<0.01 compared with 43°C group.

**Figure 7:**
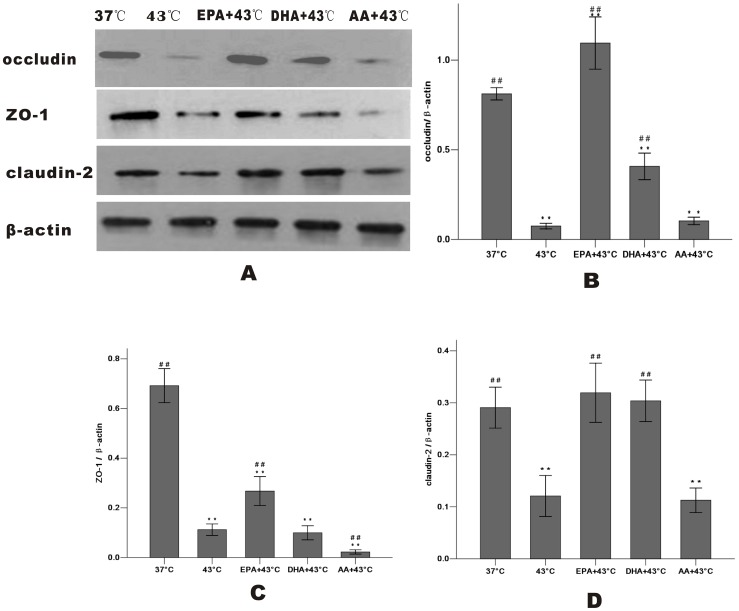
Effect of PUFAs on TJ protein expression in the membrane fraction after heat stress. Cells were cultured for 24(37°C group and 43°C group) or with PUFAs pre-incubation for 96 h. TJ proteins in the membrane fraction were shown (A): occludin (B), ZO-1 (C) and claudin-2 (D). Results were reported as means ± SD from 3 independent experiments. Values were normalized to β-actin. * P<0.05, ** P<0.01 compared with 37°C group.^ #^ P<0.05,^##^ P<0.01 compared with 43°C group.

**Figure 8:**
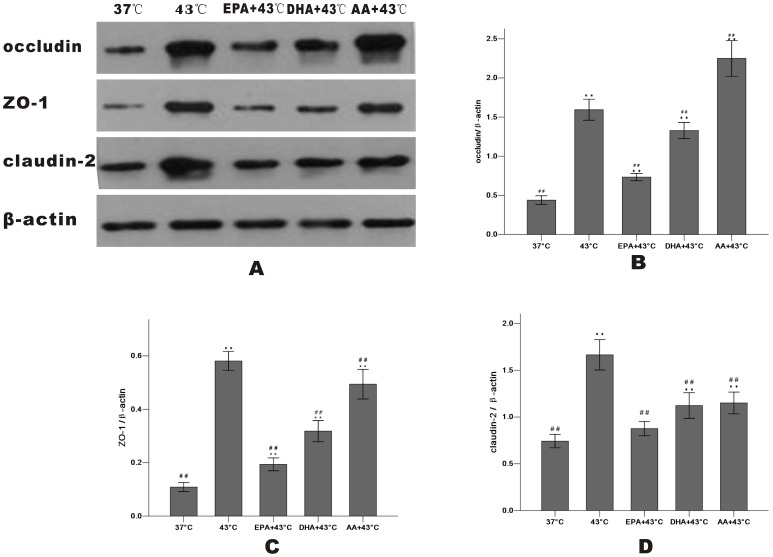
Effect of PUFAs pretreatment on TJ protein expression in the cytosol fraction after heat stress. Cells were cultured for 24(37°C group and 43°C group) or with PUFAs pre-incubation for 96 h. TJ proteins in the cytosol fraction were shown (A): occludin (B), ZO-1 (C) and claudin-2 (D). Results were reported as means ± SD from 3 independent experiments. Values were normalized to β-actin. * P<0.05, ** P<0.01 compared with 37°C group.^ #^ P<0.05, ^##^ P<0.01 compared with 43°C group.

After heating, Western blot analysis revealed that treatment with EPA significantly increased occludin and ZO-1 expression of whole cells, while DHA was less effective and AA wasn't. There is no change of the total amount of claudin-2 (**Fig. 6**). The levels of occludin, ZO-1 and claudin-2 after heat treatment at 43°C for 1 h were markedly decreased in the membrane fraction and incresed in the cytosol compared with the 37°C group, indicating that they dissociated from the membrane and were translocated to the cytosol. In the cells pretreated with EPA, occludin expression in the membrane increased and decreased markedly in the cytosol compared with the 43°C group. EPA also inhibited the release of ZO-1 and claudin-2 into the cytosol as DHA did occludin and ZO-1 slightly (**Fig. 7** **and** **Fig. 8**).

**Figure 9:**
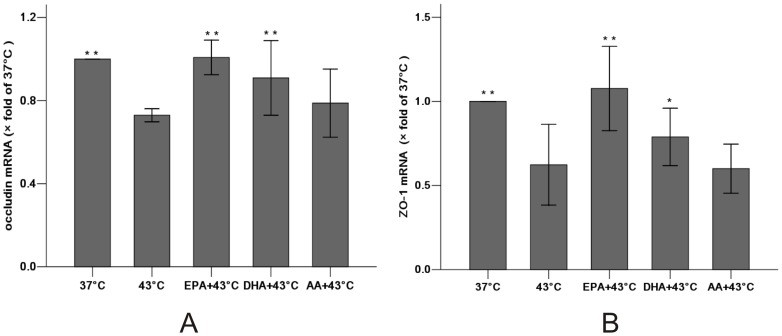
Effect of PUFAs pretreatment on the gene expressions of occludin (A) and ZO-1 (B) after heat stress by Real-time PCR. After pre-incubation with PUFAs or not (37°C group and 43°C group) for 96 h, Caco-2 monolayers were harvested 24 hours after 1 h of heat exposure. Expression of mRNA was normalized with GAPDH mRNA expression. Values were normalized to 37°C group (37°C set to 1). Results were reported as means ± SD from 3 independent experiments. N = 3 per group.* P<0.05, ** P<0.01 compared with 43°C group.

Similarly, EPA significantly increased mRNA of the heat stress-induced occludin (1.01±0.03 vs. 0.73±0.01 compared with the 43°C group, *P*<0.01) and ZO-1 (1.08±0.10 vs. 0.62±0.10, *P*<0.01). In contrast, DHA demonstrated a significant increase in occludin (0.91±0.07, P<0.01) and a modest increase in ZO-1 (0.79±0.07, P<0.05) compared with the 43°C group while AA did not result in a significant effect on either (**Fig. 9**). These results suggest that EPA significantly reduced the effects on TJ protein expression.

### EPA prevents impairment of TJ proteins induced by heat exposure

**Figure 10:**
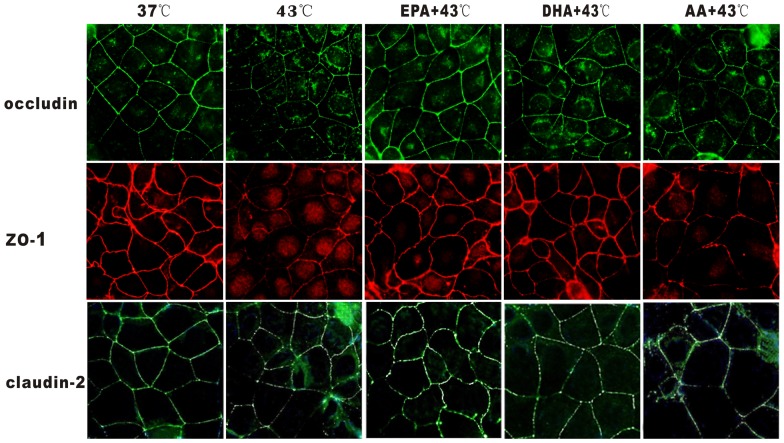
Effect of PUFAs on junctional localization of TJ proteins by immunofluorescence. Cells were pre-incubated with PUFAs or without (37°C group and 43°C group) for 96 h with heat exposure for 1 h, and cultured for 24 hours. Results were reported from 3 independent experiments. Magnification was 400×.

The effect of PUFAs on heat-induced junctional localization of occludin and ZO-1 was determined by immunostaining (**Fig. 10**). In the control group at 37°C, occludin, ZO-1 and claudin-2 presented a continuous band of cells encircling the apical cellular junctions, which would be typical for TJ proteins. Heat exposure under 43°C for 1 h caused a pronounced disruption in junctional localization and adjacent diffuse of TJ proteins staining, characterized by decreased intensity staining and marked discontinuity localized to the structures of intercellular junctions. In the EPA group, the localization and intensity of TJ proteins were more similar to the 37°C cells. In contrast, the localization and intensity of TJ proteins changed only slightly in the DHA group but did not change significantly in the AA group. These findings indicate that EPA can effectively prevent the heat induced localization of TJ proteins.

### EPA pretreatment prevents heat stress-induced morphology disruption of TJ

**Figure 11:**
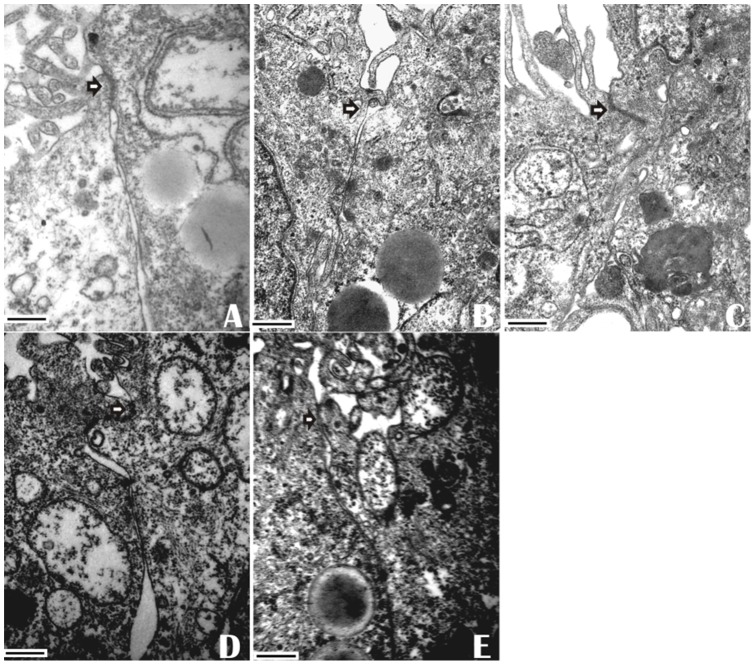
Effect of PUFAs on morphological ultrastructure of tight junction induced by heat stress. Caco-2 cell monolayers were pre-incubated without (**A:** 37°C group and **B:** 43°C group) or with EPA (**C**), DHA (**D**) or AA (**E**) with heat exposure for 1 h. Images were acquired by transmission electron microscopy after culturing for 24 h. Data are representative of 3 independent experiments. Arrows indicate tight junctions. Scale bars  = 500 nM.

Heat exposure resulted in the disruption of TJ ultrastructure in Caco-2 monolayers. In the 37°C control (no PUFAs added) Caco-2 monolayers, tight junctions were intact between the adjoining cells. After heat exposure (43°C for 1 h), TJs became markedly “open” with shortening of the strand length between the cells. TJ membranes lost fusion and had less electron-dense material. In EPA-incubated cells, the TJ strands displayed intact ultrastructure. However, DHA -treated cells had non-continuous TJ strands. AA treatment only slightly alleviated the change of tight junctions (**Fig. 11**). These results demonstrated that EPA was more effective than DHA and AA in attenuation of the distortion of TJ structure induced by heat exposure.

### PUFAs alter fatty acid composition of membrane phospholipids

Treatment with PUFAs resulted in incorporation of fatty acids into the epithelial cell membrane. EPA, DHA and AA supplementation each enriched their own composition in the membrane (P<0.01 for all). EPA and DHA supplementation did not affect AA composition. Similiarly, treatment with AA didn't significantly change the lipid composition of n-3 PUFAs (P>0.05) (**Table 1**).

**Table 1:** Fatty acid composition of membrane microdomains from control cells and PUFAs – treated cells.

	control	EPA	DHA	AA
EPA (C20:5, n3)	3.61±0.05	15.41±1.31**	3.84±0.07	3.58±0.09
DHA (C22:6, n3)	0.41±0.05	0.47±0.04	3.27±0.11**	0.39±0.04
AA (C20:4, n6)	5.79±0.12	5.37±0.12	5.53±0.09	35.66±1.32**

Caco-2 cells were pre-incubated without (control) or with EPA, DHA or AA for 96 h. Fatty acid composition was analyzed. The results were expressed as compensated area normalization. Results were reported as means ± SD from 3 independent experiments. * P<0.05, ** P<0.01 compared with control group.

## Discussion

In this study, it has been demonstrated that the increase of temperature from 37°C to 43°C damages the intestinal epithelial TJ barrier in Caco-2 monolayers and the addition of EPA can prevent the heat-induced dysfunction of intestinal TJ barrier, while DHA may do so to a lesser extent and AA shows no significant effect.

In the intestinal lumen, tight junctions maintain a barrier to keep unwanted substances out of the circulation. Under heat stress, the insult to the integrity of the intestinal wall leads to tight junction opening, promoting the massive passage of substances such as endotoxins into the internal environment so that the liver cannot remove these substances from the circulation. Elevated endotoxin levels in the systemic circulation are indicative of increased gut permeability[18]. Endotoxin levels in the blood increase in heatstroke patients at a mean rectal temperature of 42.1°C [27], [28]. It has been shown that heat stress can open tight junction leading to increasing intestinal permeability in rat models [19]. In cases of hyperthermia or excessive exercise, elevated body temperature above 40°C impairs intestinal epithelial barrier function [20]. In this study, our results also found that increasing temperature (37–43°C) reduces TEER and increases functional permeability for the large molecules of HRP in Caco-2 monolayers, indicating the physical impairment of tight junctions. These data showed the higher the temperature, the lower the TEER and the higher HRP flux. Furthermore, hyper-temperature of 43°C is harmful, since large quantity of cells were necrotized at 2 h, and were almost all dead at 5 h (data not shown). Consequently, heat stress under 43°C for 1 h were chosen in our sduty. Breakdown of tight junction integrity allows the entrance of endotoxins into the circulation, which is associated with an increased production and release of inflammatory cytokines and triggers host immune responses [21], [22].

Integral membrane proteins of tight junctions include the transmembrane proteins occludin and claudins, and the cytoplasmic adaptor proteins such as ZO-1. These proteins are important in the establishment and regulation of intestinal barrier function [23]. It is well known that alteration of TJ proteins directly regulates intestinal permeability [24]. This damaged paracellular permeability is associated with decreased expression and distribution of these proteins [25]. Decreased ZO-1 contributes to the disturbance in the intestinal TJ complex barrier. Besides playing a crucial role in the sealing of TJ, occludin regulates selective paracellular permeability [26], [27]. In the digestive tract, a reduction in or abnormal distribution of occludin is associated with the progression of tumor [28]. Previous studies have shown that this may due to the damage of the cell-cell adhesion mechanism in which TJ proteins interact directly with actin filaments and alpha-catenin [29]. Temperature elevation of 37–41°C is associated with a compensatory increase in the TJ protein occludin, which is directly correlated with a significant increase in TEER and TJ strands of the intestinal epithelial barrier, and no change in claudin-3 [30]. There is also a progressive decrease in ZO-1 at 24 h after 43°C for 30 min of heat exposure. Moreover, Li et al. found that expression of claudin-1 and claudin-4 did not appear to be influenced by DHA and EPA treatment [10]. For these reasons, we chose occludin, ZO-1 and claudin-2 to represent typical TJ proteins in our study. However, in our study, when the temperature reached 43°C, the occludin protein expression decreased dramatically compared with 41°C and was showed no significant difference compared with the 37°C group. The increase in occludin expression seen in the increase of temperature from 37°C to 41°C was attributable to a progressive increase in mRNA transcription and new protein synthesis but not to a decrease in protein degradation [19]. A compensatory increase in expression of occludin by modest heat exposure from 37–41°C results in enhancement of the TJ barrier, because inhibition of occludin produces a decrease in TEER and TJ permeability during heat exposure [17]. Our data indicated that exposure to 43°C halted the increase and resulted instead in a relative decrease in both transcription and expression of the occludin. In contrast, there is a significant decrease in ZO-1 expression at temperatures from 37°C to 43°C, which was associated with heat stress-induced increase in TJ permeability [30], [31].

Moreover, heat exposure induced the translocation of occludin from the memberane into the cytosol. The ratios of membrane-bound to cytosolic tight junction proteins correlated with the development of TEER in epithelial cells [32]. Because the function of occludin in tight junctions requires phosphorylation, the TJ barrier breaks down when occludin is dephosphorylated, which correlates with its transfer from the tight junction into the cytoplasm [33]. ZO-1 and claudin-2 expression was also found to be less in the membrane and greater in the cytosol. As a result, we conclude that expression and redistribution of TJ proteins, provides the molecular basis for barrier impairment after heat stress.

Although the mechanism by which n-3 PUFAs alleviate these heat-induced permeability defects and epithelial barrier dysfunction remains incompletely understood, several recent studies have provided some insights into the possible mechanism involved. Intestinal permeability is regulated either directly through alteration of TJ proteins, or indirectly through effects on the cytoskeleton [1]. It has been demonstrated that n-3 PUFAs alleviate the changes in tight junction structure and modulate TJ protein expression [31]. In a study of ulcerative colitis (UC) in a rat model, EPA and DHA were found to attenuate the disruption of TJ structure by elevating expression and preventing redistribution of tight junction proteins such as occludin and ZO-1 [11].

Many previous studies have shown that EPA is more effective than DHA in alleviating the changes in tight junction structure and in modulating TJ protein expression. In endothelial cells EPA pre-treatment is associated with improved TJ function through an increase in the expression of occludin and ZO-1, but AA exerts the opposite effect [13], [34]. Similarly, High-EPA diets augment the level of cerebral occludin protein in rats [35]. Firstly, NF-κB signaling possibly modulates the alteration of change of TJ proteins. EPA and DHA appear to have anti-inflammatory effects by reducing the secretion of cytokine via the NF-κB signaling system [36], [37], while n-6 PUFA showed no such effect [38]. EPA is more potent than DHA in modulating NF-κB p65 DNA binding by decreasing IKK expression [39]. The inhibition of the NF-κB signaling system results in increased expression and decreased redistribution of occludin and ZO -1 at cell junctions [40]. Similarly, in this study, EPA was found to more effectively improve occludin and ZO-1 expression and inhibit the translocation of occludin, ZO-1 and claudin-2 from membrane into the cytosol in intestinal cells after heat treatment. This was associated with increased intestinal TJ barrier function. Secondly, the epithelial barrier function was also mediated by the specialized lipid composition of the lipid raft fractions [10]. Incorporation of PUFAs into the cell membranes mediates the subcellular distribution and structure of TJ proteins [41]. It has previously been described that EPA pre-treatment in particular is more effective than DHA in supporting the permeability of tight junctions induced by the cytokines through increasing fatty acid and phospholipid composition in lipid raft fractions. This may protect TJ function from cytoskeleton reorganization and morphological changes of tight junction. AA does not show this effect [10], [42]. Thirdly, eicosanoids, which maily derive from AA, are proposed as mediators of induced cytoskeleton reorganization and changes in TJ function [24], [43], inducing a decrease in endothelial permeability. EPA and DHA have been shown to ameliorate the immune response by reducing the production of pro-inflammatory mediators, including eicosanoid mediators from AA [44], [45]. EPA and DHA treatment could decrease eicosanoids in the rafts [10]. In addition, metabolites of eicosanoids produced by EPA have a different structure from those made from AA and are much less biologically active [46]. Our results showed that treatment with EPA, more so than with DHA, markedly enriched themselves in the membranes of Caco-2 cells. Therefore, EPA more effectively modulates the integrity of cell membrane permeability. Thus, these data provide additional support for the notion that EPA is more effective than DHA in stimulating basal resistance, ameliorating permeability, and attenuating the distortion of TJ structure by heat stress. The mechanism involved needs to be further studied.

In contrast, other studies have shown EPA and DHA impaired barrier integrity in vitro. In fact, effects of various factors affecting intestinal permeability in monolayer cells vary with different PUFA concentrations and incubation times. These negative results may be explained by shorter incubation times with PUFAs (24 h) with either a decrease in or no change of TEER [13]. However, a time period of 96 h in Willemsen's study reported enhanced effect of PUFAs on tight junction function in endothelial cell culture [42]. Our results also showed that EPA did not significantly increase TEER until 96 h compared with the control group. In our study, the most effective protection in Caco-2 cells was observed after incubation with 50 μM PUFA for up to 96 h (data not shown). This replicates the findings by Usami et al. that 50 μM EPA is beneficial to TEER and fluorescein sulfonic (FS) permeability compared with other concentrations of PUFAs. Furthermore, incubation with a higher concentration of PUFA (100–200 μM) reduced TEER, and increased FS permeability of Caco-2 cells [47], [48]. Moreover, adding vitamin C and vitamin E, (with the same amount added to the control group to control for the effect of PUFAs [42]) prevented lipid peroxidation-induced cytotoxicity during longer incubation times with PUFAs [49].

Our results indicate for the first time that EPA attenuates the increase of intestinal epithelial TJ permeability induced by heat stress. Moreover, our results also show that EPA modulates the expression of occludin and ZO-1 in whole cells and the translocation of the TJ proteins from the membrane to the cytosol, which are responsible for ameliorating heat-induced decrease in TEER and increase in HRP flux. These results indicate that EPA pretreatment may prevent dysfunction of tight junction permeability from heatstroke. EPA has already been used as a treatment for hyperlipidemia and cardiovascular disease in Japan [50]. Other studies have demonstrated that dietary EPA can be safely administered and be beneficial for improving clinical outcomes in critically ill patients [51], [52]. Because of its support of the intestinal epithelial barrier, pretreatment with EPA may be effective in preventing the occurrence of heatstroke in high-risk populations undergoing heat stress. Further experiments are still needed to examine the exact mechanism of the beneficial role played by EPA in improving gut integrity in vivo.
